# Midterm surgical results of the lamina with spinous process in treating one-level thoracic or lumbar tuberculosis: a retrospective study

**DOI:** 10.1038/s41598-020-79209-x

**Published:** 2020-12-16

**Authors:** Weiyang Zhong, Xinjie Liang, Ke Tang, Tianji Huang, Xiaoji Luo, Zhengxue Quan

**Affiliations:** 1 Department of Orthopedic Surgery, The First Affiliated Hospital of Chongqing Medical University, Chongqing, China; 2 Department of Pain Management, The First Affiliated Hospital of Chongqing Medical University, Chongqing, China

**Keywords:** Tuberculosis, Neurosurgery

## Abstract

A retrospective study investigated the results of the lamina with spinous process (LSP) as a bone graft in one-level thoracic or lumbar spinal tuberculosis with the one-stage posterior approach of debridement, fusion and internal instrumentation. Data from 35 patients from January 2013 to December 2015 were analysed. Surgery time, blood loss, hospitalization time, drainage volume, and follow-up (FU) duration were recorded. The visual analogue scale (VAS), Oswestry Disability Index (ODI), erythrocyte sedimentation rate (ESR), C-reactive protein (CRP), American Spinal Injury Association (ASIA) grade, segmental angle, and bone fusion were compared between preoperative and final FU. All of the patients were followed up for a mean 43.90 ± 10.39 months. The mean age, surgery time, blood loss, hospitalization time, hospital cost and drainage volume were 33.65 ± 11.06 years, 182.40 ± 23.82 min, 280.80 ± 76.82 mL, 14.05 ± 3.58 days, 74,382.00 ± 11,938.00 yuan, and 340.00 ± 167.20 mL, respectively. VAS and ODI were significantly improved at the final FU. The ESR and CRP recovered to normal. The mean angle of 24.35 ± 5.74°preoperatively showed a significant difference between 1 week, postoperatively and final FU. Although there were the loss of angle at final FU comparing with the 1 week postoperatively, it still maintain the good alignment and the segmental stability. All patients achieved bony fusion with a mean time of 12.90 ± 3.91 months. In conclusion, the LSP as a structural bone graft is reliable, safe and effective for segmental stability reconstruction, which could be one choice for surgical management of thoracic or lumbar spinal TB.

## Introduction

Spinal tuberculosis (TB) is a common extrapulmonary form of an ancient infectious disease. TB kills 1.81 million people per year in Asia, and 78% of new cases reported each year are in China, according to the World Health Organization (WHO)^1,2^. Spinal TB is especially dangerous, resulting in bone destruction, spinal deformity, and/or even paraplegia^3,4^. Hence, more attention should be paid to spinal TB. Usually, although anti-TB drugs and other management are effective, there are problems with its treatment because the principles of drug treatments differ significantly according to our understanding of the treatment of pulmonary TB, the emergence of drug resistance and spinal TB in HIV-positive patients^5,6^. Fortunately, surgical management can be effectively performed when TB results in spinal deformity and neurological deficits^7,8^. Surgery is considered one of most effective methods of curing spinal TB.

After debridement and decompression, many interbody bone grafts have been used to regain spinal stability, such as iliac crest, or ribs or fibula grafts or titanium mesh cages, which have their advantages and limitations. Iliac crest or fibula grafts as the "gold standard", could achieve a high rate of bone healing which complications at the donor site, such as persistent pain, haematoma, and unhealed wounds can also occur. The use of allografts is associated with the risk of disease transmission. Autologous bone grafts with a titanium mesh cage have been widely used which have the problems such as subsidence, stress occlusion, and radiation opacity^9–11^. To date, no study has reported the use of lamina with spinous process (LSP) as a bone graft in the surgical treatment of one-level thoracic or lumbar spinal TB. This study aimed to investigate the clinical effect of LSP as a bone graft for restoring spine stability.

## Materials and methods

### Patient population

This study was approved by the Institutional Review Board of the First Affiliated Hospital of Chongqing Medical University and conducted in accordance with the Declaration of Helsinki. All participants provided written informed consent before their data were stored in our hospital database and used for study purposes. The treatment plans were all developed while providing routine clinical care. When communicating with patients before surgery, the advantages and disadvantages of the possible approaches were fully explained to the patients and their family members, so the patients could choose the right treatment for themselves.

From January 2013 to December 2015, in the spine unit of our department, the data from 35 patients with one-segment thoracic or lumbar spinal TB were reviewed retrospectively. The surgery procedure was performed by the same spine team for all patients.

The inclusion criteria were adult thoracic or lumbar spinal TB, one-stage posterior approach, and internal instrumentation and reconstruction. The patients were indicated for surgery because of the following: increasing kyphosis, neurological deficits, bone destruction affecting spine stability, and the bone damage of the infected vertebral body did not exceed 1/2 the vertebral height of the thoracic spine and did not exceed 1/3 the vertebral height of the lumbar spine. The exclusion criteria were active pulmonary TB, spine fractures and spine cancer.

### Preoperative management

Once the clinical diagnosis was made, anti-tuberculosis chemotherapy was given immediately. Anti-TB drugs in the HREZ standard programme include isoniazid (5–10 mg/kg/day), rifampicin (10 mg/kg/day), ethambutanol (15 mg/kg/day) and pyrazinamide (25 mg/kg/day), which were administered 3–4 weeks before surgery^2,10^. Surgery was performed when ESR, c-reactive protein (CRP), and body temperature recovered to normal or decreased significantly, or when ESR decreased below 60 mm/h and CRP decreased gradually. When the neurological dysfunction gradually worsened and anti-TB drug therapy failed, we shortened the duration of drug therapy and considered emergency surgical treatment.

### Surgical techniques

**Figure 1:**
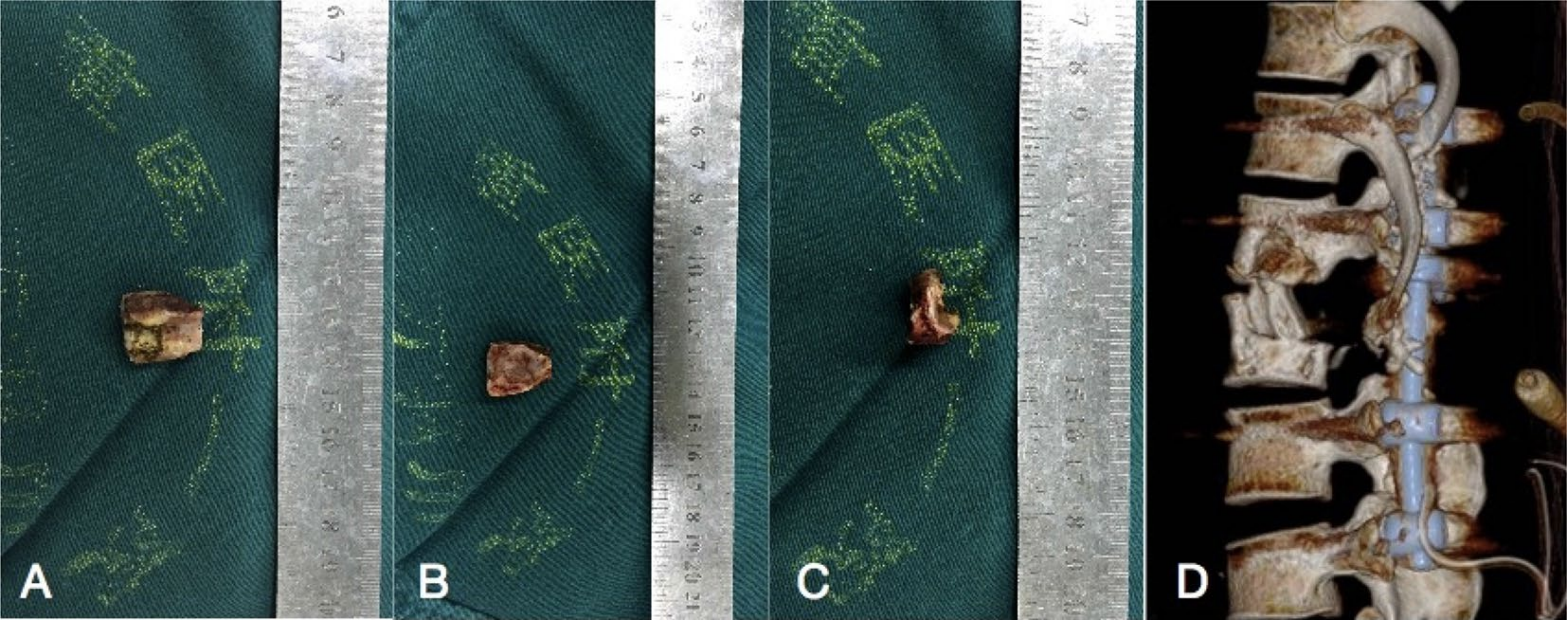
Photographs of one LSP before (**A**–**C**) and after implantation (**D**).

After the administration of general anaesthesia, the patients were placed in the prone position. Posterior segments of the spine, such as the lamina and facet joints, were exposed through a median incision. Pedicle screw fixation was performed according to imaging and c-arm X-ray results to ensure accuracy. The entire LSP was cut off using a sharpened drill or ultrasonic osteotome, and then one of the facet joints was removed for decompression and radical debridement. The LSP was trimmed by a bone knife, a sharpened drill, or an ultrasonic osteotome to obtain a suitable and three-cortical-sided bone graft (Fig. 1). Depending on the remaining space, one LSP was trimmed suitably, before the implantation of LSP, a few of autologous granular bones were filled in the bone defect and afterward, the instrumentation was locked with force. Gelatine was used locally mixed with 1.0 g streptomycin and 0.2 g isoniazid. Negative pressure drainage was performed, and the collected specimens were sent for bacterial culture and pathological findings.

### Postoperative care

After the surgery, the oral chemotherapy regimen (12 HREZ/12-18HRE) for the patients must be followed up strictly with the physician, paying close attention to the side effects of the anti-TB drugs and adjusting the drugs and treatment in a timely fashion when they occurred. Walking training under the guidance of a rehabilitation therapist was started 1 week after the surgery. Clinical and imaging examinations for all of the patients were performed at 1 week, 3 months, 6 months, 12 months and then annually after surgery.

### Outcome assessment

Perioperative and follow-up (FU) observations were collected from the patient data: (1) the operation time, surgery haemorrhage, hospital stay, drainage, FU time and bone fusion time. (2) Segmental angle. According to the Cobb method^11^, the segmental angle was defined as the angle formed between the superior endplate of the upper vertebral body and the inferior endplate of the lower vertebral body. (3) ESR and CRP. (4) VAS and ODI. (5) Neurological function assessed by American Spinal Injury Association (ASIA) grade. The bone fusion was assessed using the criteria of Bridwell et al. with the X-ray and CT when necessary. All radiographic data and measurements in our study were reviewed by one senior spine surgeon and one senior radiologist.

### Statistical analysis

The statistical analysis was performed using Statistic Analysis System (SAS Institute Inc., Cary, NC, USA). The results are expressed as the mean ± SD and Student’s t-test was performed. Differences with P values < 0.05 were considered statistically significant.

## Results

**Figure 2:**
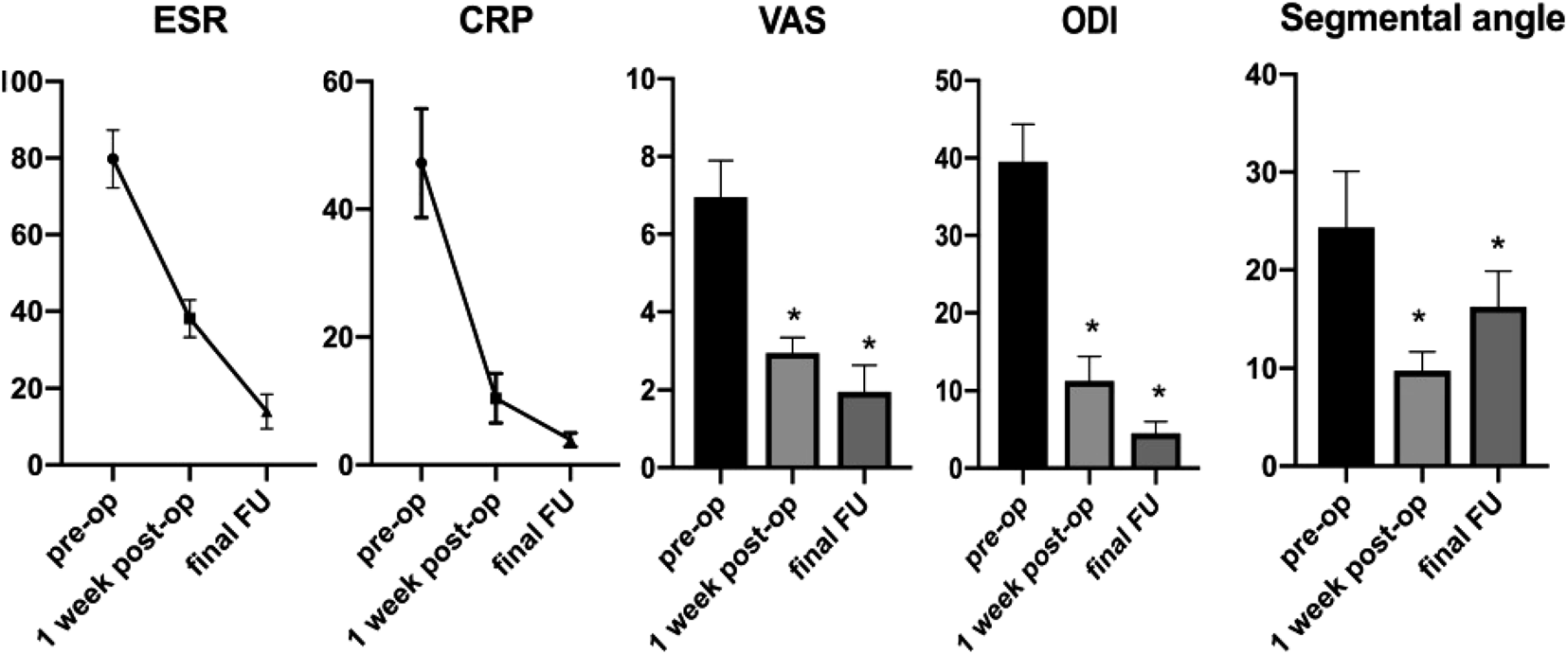
Presentation of ESR, CRP VAS, ODI, Segmental angle of preoperation, 1 week postoperation, final follow-up.

All of the patients were followed up for a mean 43.90 ± 10.39 months; the mean age, surgery time in minutes, blood loss, hospitalization time, hospital cost and drainage volume were 33.65 ± 11.06 years, 182.40 ± 23.82 min, 280.80 ± 76.82 mL, 14.05 ± 3.58 days, 74,382.00 ± 11,938.00 yuan, and 340.00 ± 167.20 mL, respectively (Table 1). The CRP, ESR, VAS, and ODI were decreased significantly at the final FU relative to their preoperative values (P < 0.05) (Table 2, Fig. 2). The neurological impairments were improved significantly at the final FU using ASIA grading (Table 2).

**Table 1:** Patient characteristics.

No. of patients (n)	35
Male/female (n)	19/16
Mean age (years)	33.65 ± 11.06
Disease course (months)	17.95 ± 8.93
Hospital stay (days)	14.05 ± 3.58
Hospital cost (yuan)	74,382.00 ± 11,938.00
Surgery time (min)	182.40 ± 23.82
Blood loss (mL)	280.80 ± 76.82
Drainage (mL)	340.00 ± 167.20
Mean fusion time (months)	12.90 ± 3.91
Follow-up (months)	43.90 ± 10.39

**Table 2:** Clinical and radiographic outcomes.

	Before treatment	1 week post-op	The final FU	P
ESR (mm/h)	79.75 ± 7.55	38.10 ± 4.84	13.95 ± 4.50	< 0.0001
CRP (mg/L)	47.20 ± 8.51	10.45 ± 3.87	3.95 ± 1.05	< 0.0001
VAS	6.95 ± 0.94	2.95 ± 0.39	1.95 ± 0.69	< 0.0001
ODI	39.95 ± 4.84	11.25 ± 3.19	4.50 ± 1.54	< 0.0001
Segmental angle (°)	24.35 ± 5.74	9.75 ± 1.94	16.25 ± 3.64	< 0.0001
**ASIA**				
A				
B	2			
C	8	5		
D	25	30	2	
E			33	< 0.0001

**Figure 3:**
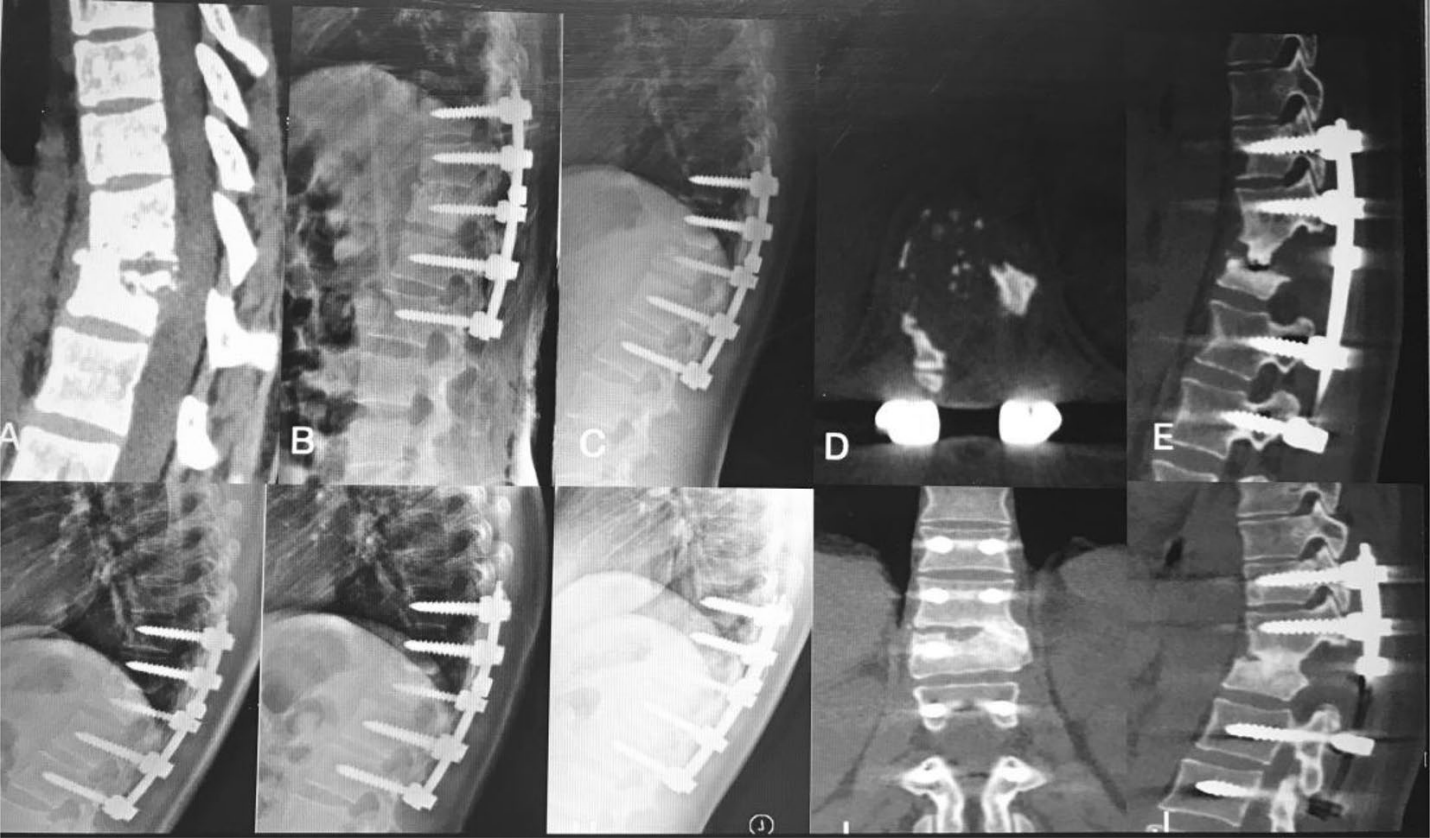
A 22-year-old female patient with thoracic spinal tuberculosis (T11–12) underwent posterior debridement and decompression combined with instrumentation. (**A**) Preoperative computed tomography (CT) shows bone destruction of the T11–12 vertebrae and compression of the spinal cord. (**B**–**E**) The 1-week and 6-month postoperative X-rays and 6-month postoperative CT show the correction was maintained, but the bone was not fully healed. (**F**,**G**) The 1-year and 2-year postoperative X-ray show the kyphosis correction had been lost. (**H**–**J**) At 5-year FU, plain X-ray and CT showed solid bone fusion.

**Figure 4:**
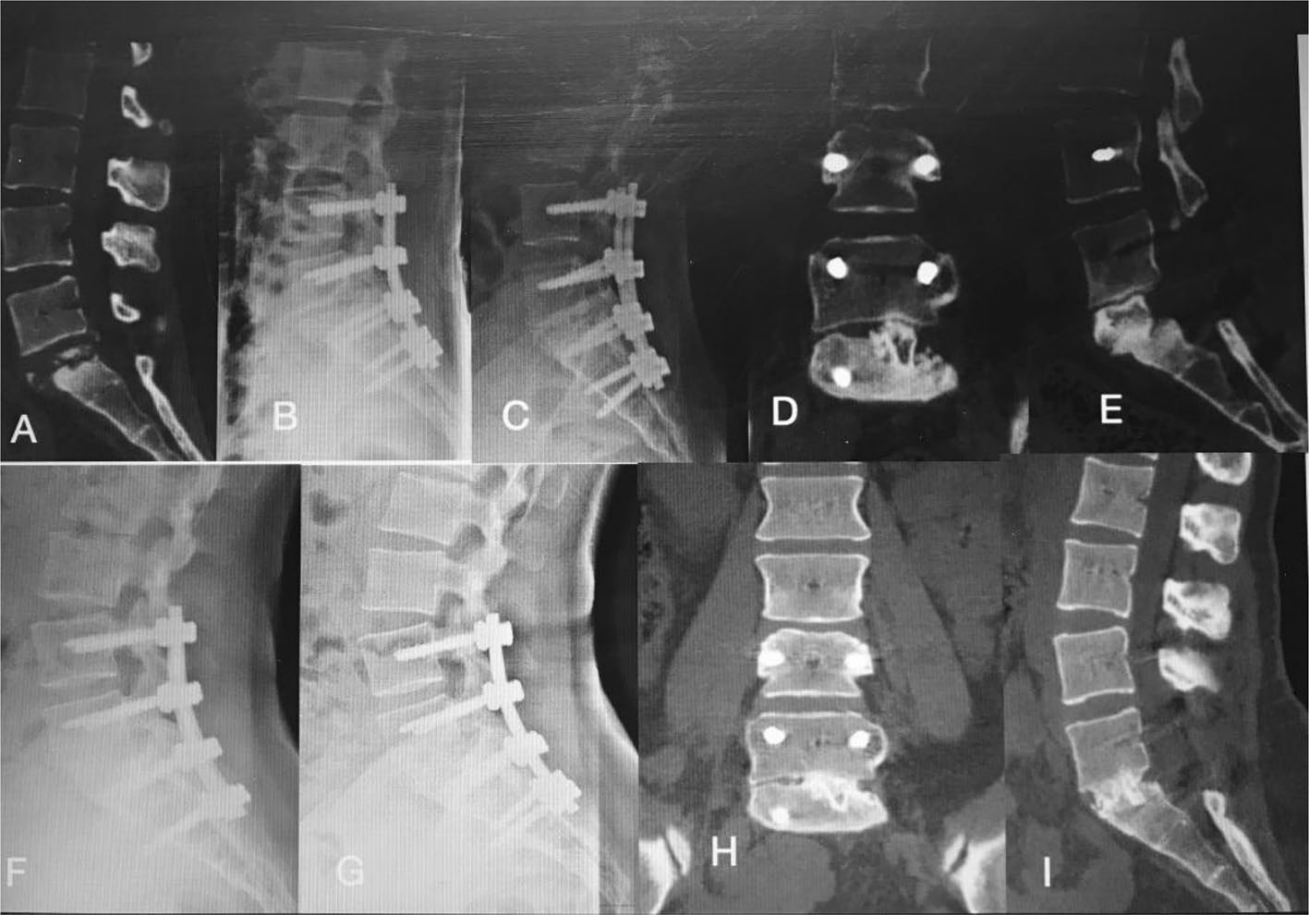
A 28-year-old female patient with lumbosacral spinal tuberculosis (L5-S1) underwent posterior debridement and decompression combined with instrumentation. (**A**,**B**) Preoperative computed tomography (CT) shows bone destruction of the L5-S1 vertebrae and compression of the nerve, and 1-week X-ray shows the correction was maintained. (**C**–**E**) The 6-month postoperative X-ray and 6-month postoperative CT show the correction was maintained, but the bone was not fully healed. (**F**) The 1-year and 2-year postoperative X-rays show the kyphosis correction has been maintained. (**G**–**I**) At 4-year follow-up, plain X-ray and CT show solid bone fusion.

**Figure 5:**
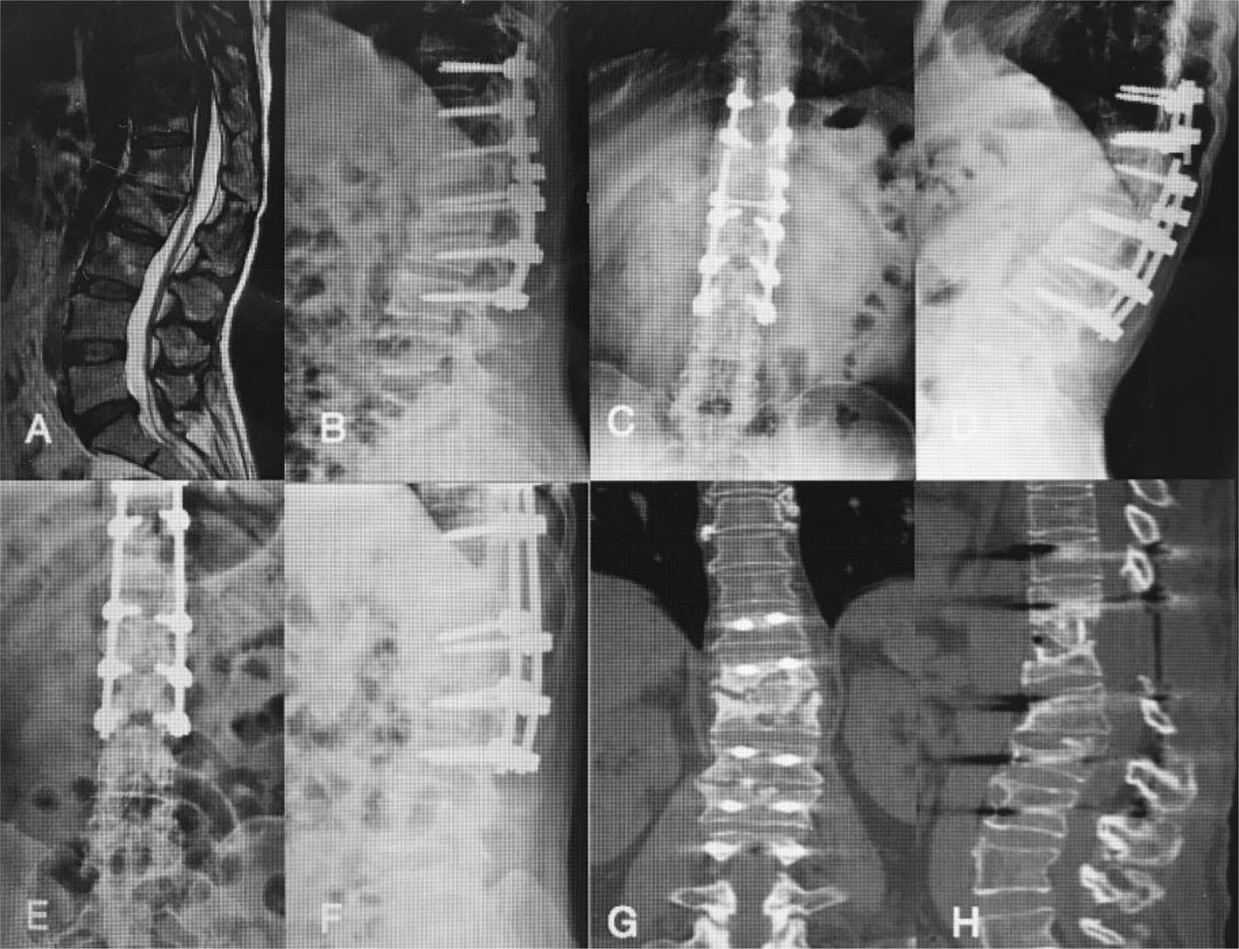
A 51-year-old woman with thoracic spinal tuberculosis (T11–12) underwent posterior debridement and decompression combined with instrumentation. (**A**,**B**) Preoperative computed tomography (CT) shows bone destruction of the T11–12 vertebrae and compression of the spinal cord. (**C**,**D**) The 2-year postoperative X-ray and CT demonstrate the bone was unhealed, and a rod fracture was observed. (**E**–**H**) The revision surgery was performed with an iliac graft. X-ray and CT show the good location of the bone graft.

The thoracic spinal TB was well cured and all patients achieved bony fusion at a mean 12.90 ± 3.91 months. The mean angle of 24.35 ± 5.74° preoperatively showed a significant difference between 1 week postoperatively and final FU. Although there were the loss of angle at final FU comparing with the 1 week postoperatively, it still maintain the good alignment and the segmental stability (Figs. 3, 4). One case of rod fracture was observed 2 years postoperatively, and the revision surgery was performed with an iliac graft (Fig. 5). Some postoperative complications occurred, such as water-electrolyte imbalance (two cases) and superficial infection (two cases).

## Discussion

With the rising incidence of TB, there are more than eight million new cases of tuberculosis each year, with it remaining a leading infectious disease cause of death globally^1–3^. Spinal TB is the most common and severe form of osteoarticular TB. Formal adequate and long-term anti-tubercular drugs, bed rest and supportive nutrition are necessary in the management of spine TB^4–6^. Despite the efficacy of conservative regimens, surgical treatment is still recommended for patients with bone destruction, sequestration, a paraspinal or spine canal abscess, or neurological impairment. Surgery, such as the single-stage anterior approach, can be challenging to apply because of the need to overcome severe trauma such as massive blood loss, a longer operation time, and more serious complications. A posterior pedicle screw system with bone grafting and correction of the kyphotic deformity has been widely applied for stabilization, effectively treating thoracic or lumbar spinal disorders. Hence, the principles of management of spinal TB have been modified to allow them to become more accurate and individualized^7–13^.

After surgical debridement and decompression of the spinal cord or nerves, a variety of intervertebral bone grafts can be used to restore the stability of the anterior and middle columns. Iliac crest or fibula grafts, considered the "gold standard", could achieve a high rate of bone healing. However, complications at the donor site, such as persistent pain, haematoma, and unhealed wounds, can also occur. The use of allografts can avoid complications, but their use is associated with an increased rate of graft failure and the risk of disease transmission. Autologous bone grafts with a titanium mesh cage have been widely used, and they have a high bone fusion rate. However, problems such as subsidence, stress occlusion, and radiation opacity can affect surgical planning^14–20^. Although the transverse process had advantages of reducing trauma, shortening hospital stay, the bone defects caused by the lumbar spine TB are larger than these in thoracic spine which the transverse processes of lumbar spine have deeper anatomical characteristics limited by difficulty in being exposed and which the mechanical requirements of lumbar spine are higher than thoracic spine^21–24^. Therefore, this study aimed to find a new method of bone grafting to provide support and promote bone fusion to reduce the incidence of complications.

The use of the LSP as the bone graft has the following advantages. First, in our study, the surgery time in minutes, blood loss, hospitalization time, hospital cost and drainage volume were 182.40 ± 23.82 min, 280.80 ± 76.82 mL, 14.05 ± 3.58 days, 74,382.00 ± 11,938.00 yuan, and 340.00 ± 167.20 mL, respectively. Compared with the iliac crest or fibula graft used in previous studies^17–24^, the use of the LSP could reduce trauma and bleeding, shorten surgical and hospitalization times, decrease postoperative drainage volumes, and reduce postoperative complication rates. The LSP are present in the surgical exposure area during the posterior approach, which can reduce bleeding and trauma. Second, in our study, all patients achieved bony fusion at a mean 12.90 ± 3.91 months. The mean angle of 24.35 ± 5.74° preoperatively showed a significant difference between 1 week postoperatively and final FU. Although there were the loss of angle at final FU comparing with the 1 week postoperatively, it still maintain the good alignment and the segmental stability. Furthermore, the VAS and ODI were also improved during FU, and thus, the patients recovered soon after surgery and VAS and ODI were significantly improved at the final FU. Hence, the use of LSP could achieve biomechanical stability and satisfactory clinical efficacy. Although the LSP could provide good support, strength, and fusion properties, the bony fusion time was long compared with other bone grafts, which could have caused the segmental angle loss. One case of rod fracture was observed at 2-year FU because of the bone union.

Hence, the LSP, as an autogenous bone, has a cortical bone structure and is short on cancellous bone tissue. The use of the LSP could be suitable for supporting the bone defect space, which could ensure and maintain the segmental stability and could be one more choice for the surgerons, However, using a single LSP as a bone graft had a risk of delayed bony fusion or even nonunion, and therefore it could be suggested that longer use of a brace is necessary while the bone is not fully healed during the FU.

## Conclusion

Our study results showed that use of the LSP as a bone graft could be one choice in the surgical management of one-level thoracic or lumbar spinal TB, resulting in good bone fusion and spinal stability restoration. It is a reliable, safe, effective and structural bone grafting method. However, we declare that the retrospective nature of this small-sample study may be associated with bias. Second, although we are the first to report the use of LSP as a structural bone graft, the use of a single LSP as a bone graft had a risk of delayed bony fusion or even nonunion. Third, our study did not consider intra- and inter-observer differences, which are associated with bias. Fourth, we need to perform biomechanical tests to determine differences in strength, which could confirm that the LSP meets the biomechanical needs. In the future, prospective, randomized studies with long-term follow-up are needed.
